# Simultaneous Determination of Yeast Inhibitors 5‑HMF and Furfural in Hydrolyzed Lignocellulosic Biomass using HPLC-PDA

**DOI:** 10.1021/acsomega.5c03283

**Published:** 2025-06-02

**Authors:** Jhonatan M. P. Rocha, Giovano Tochetto, André L. Gallina, Daiane F. Ferreira

**Affiliations:** † Campus CEDETEG, Chemistry Department, Universidade Estadual do Centro-Oeste, Elio Antônio Dalla Vecchia Avenue, 838, Vila Carli CEP, 85040-167 Guarapuava, Paraná, Brazil; ‡ Post-Graduate Program in Bioenergy, Campus CEDETEG, Universidade Estadual do Centro-Oeste Campus CEDETEG, Elio Antônio Dalla Vecchia Avenue, 838, VilaCarli CEP, 85040-167 Guarapuava, Paraná, Brazil

## Abstract

The lignocellulosic biomass acid hydrolysis process, for either pretreatment or saccharification purposes, involves temperature and acidity, which can lead to carbohydrate dehydration into furfuraldehydes, such as 5-hydroxymethylfurfural (5-HMF) and furfural. Unfortunately, these compounds can reduce the biomass quality for biofuel production, potentially inhibiting yeast fermentation, which converts sugars into ethanol, leading to low yields. Given the need to control these substances, a methodology for the simultaneous determination of 5-HMF and furfural via high-performance liquid chromatography (HPLC) was developed and validated to monitor the formation of these unwanted byproducts directly after the hydrolysis process of the Hevea brasiliensis lignocellulosic matrix. The method showed adequate selectivity for both analytes. Linearity was confirmed by analysis of variance (*p* < 0.05) for 5-HMF and furfural, with excellent correlation coefficients: *R* ^2^ = 0.99984 in the 0.1–50 μg·mL^–1^ range for 5-HMF, and *R* ^2^ = 0.99956 in the 0.1–25 μg·mL^–1^ range for furfural, with low limits of detection and quantification: 0.1981 and 0.6002 μg·mL^–1^ for 5-HMF, and 0.1585 and 0.4802 μg·mL^–1^ for furfural, respectively. The method also demonstrated accuracy, with recovery rates in fortified samples between 100.7 and 104.9% for 5-HMF and 97.54 and 100.4% for furfural. Precision, divided into repeatability and intermediate precision, showed both values for RSD < 15%. Additionally, the method demonstrated robustness, maintaining expected performance when subjected to small variations. The developed method proved to be a quick, effective, and reliable approach for quantifying 5-HMF and furfural in the hydrolyzed lignocellulosic biomass, successfully applied to 43 real samples without the need for complex pretreatment and with a shorter run time and high sensitivity. This makes it suitable for routine monitoring and supports more practical, scalable, and both time and cost-effective strategies for optimizing biomass conversion and bioethanol production.

## Graphical abstract

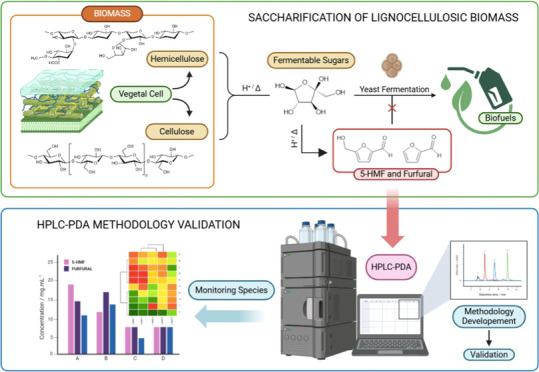

## 1 Introduction

Currently, a large portion of the energy produced by humans comes from nonrenewable fossil sources such as oil, coal, and natural gas. Due to the current energy demands, the use of these sources has been rising almost exponentially, triggering various environmental issues and climate changes caused by the massive emission of greenhouse gases from these fuels. Moreover, concerns about energy insecurity arising from dependence on nonrenewable sources, combined with their environmental consequences, have been pressuring the scientific and technological community to develop new, more eco-friendly fuel alternatives derived from renewable sources.^1,2^

The conversion of plant lignocellulosic biomass into bioethanol has become a promising alternative to produce renewable fuels, as it is widely available and rich in complex sugars that can be converted into simple sugars (such as glucose and sucrose), which, in turn, can be fermented into ethanol. However, due to the chemical nature of its complex carbohydrates, such as cellulose and hemicellulose, and the variable lignin content depending on plant source, genus, and species,^3^ some essential extra steps are required for the saccharification of this kind of matrix prior to fermentation.^2^

The saccharification of the cellulose and other carbohydrate polymers of lignocellulosic biomass generally occurs through the action of enzymes or chemical treatments involving acidification and heating.^2^ In this process (Figure 1), the glycosidic bonds between the glucose molecules and other reducing sugars that make up the cellulose are broken, with the result being free reducing sugars.^4^ Acid hydrolysis is one of the oldest and most studied methods of saccharification, capable of converting cellulose and hemicellulose into fermentable sugars.^1^ However, the high acidity and temperature of this kind of reaction can trigger the dehydration of some hydrolyzed pentoses (xylose and arabinose) and hexoses (glucose and fructose), leading to the formation of furfuraldehydes, which are undesirable for fermentation purposes, such as 5-(hydroxymethyl)-2-furfuraldehyde (5-HMF) and 2-furfuraldehyde (furfural).^4^ These substances can also undergo polymerization to form humins, significantly reducing the quality of the substrate for bioenergy purposes.^5^

**Figure 1:**
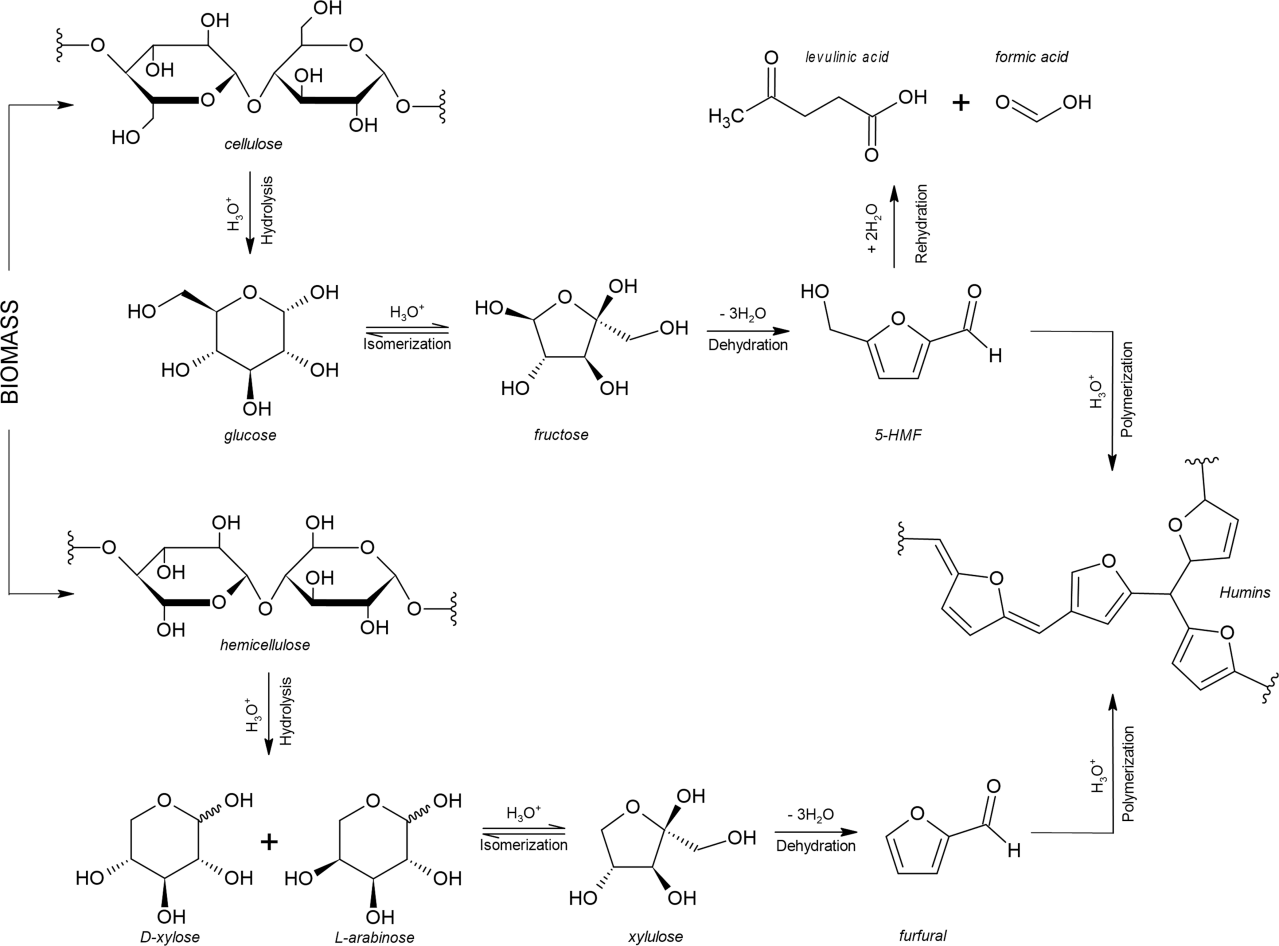
Hydrolysis of lignocellulosic biomass, formation of 5-HMF and furfural, and possible reaction pathways, as described by Mittal et al.^9^ and Sweygers et al.^5^ (Created by the authors).

On the other hand, enzymes are currently the most commonly used for the saccharification process, with several advantages. However, the large presence of lignin in lignocellulosic biomass, along with hemicellulose, makes access to cellulose, the largest source of carbohydrates from this type of biomass, very difficult. Indeed, cellulose, which is found mostly on the cellular walls of plants, is encased in a structure made of hemicellulose and lignin.^6^ As such, pretreatment steps are necessary before saccharification with enzymes, with some pretreatment methods being able to trigger the creation of the aforementioned inhibitors.^7^

5-HMF and furfural have been identified as potent dose-dependent inhibitors of yeast cell growth. Although they can act synergistically, some studies indicate that these microorganisms exhibit greater sensitivity to furfural than to 5-HMF. Taherzadeh et al.,^8^ in studies with Saccharomyces cerevisiae, reported that the addition of 4.0 g·L^–1^ of 5-HMF was able to inhibit 32% of the CO_2_ production rate, with the compound being oxidized to alcohol by the yeast at a rate of 0.14 ± 0.03 g·g^–1^h^–1^. In addition, when 5-HMF and furfural were added together, yeast growth was completely inhibited until all furfural was converted, highlighting the sensitivity of yeast to these substances.

In studies by Iwaki et al.,^10^ the authors indicate that furfural and 5-HMF induce translation repression and the accumulation of untranslated mRNAs, promoting the formation of cytoplasmic mRNP granules (stress granules) in S. cerevisiae. The combination of these two compounds intensified translation initiation repression and induced the formation of these stress granules, which serve as indicators of cellular stress during the fermentation of lignocellulosic hydrolysates.

Given the importance of minimizing these products in biomass pretreatment and saccharification processes, it is crucial to monitor these substances. Chromatography and spectrophotometry are some of the most used techniques for determining 5-HMF and furfural in complex matrices. However, only chromatography demonstrates high selectivity, reliability, and reproducibility, with high-performance liquid chromatography (HPLC) standing out.^11^ Methodologies for the determination of 5-HMF via HPLC have already been described in various matrices, primarily in honey,^12^ diverse foods,^13^ beverages,^14^ among others, as 5-HMF is one of the main indicators of food quality deterioration in carbohydrate-containing foods, in addition to being considered toxic to humans.^11^ Furfural is also associated with plant-based matrices,^15^ and methodologies have been proposed for its quantification in lignocellulosic biomass, along with 5-HMF.^16^ One of the most common detectors for these types of substances in HPLC is the photodiode array (PDA), as these substances are easily detected due to their strong absorption in the ultraviolet region between 270 and 285 nm.

In this study, given the need to monitor these substances after the biomass pretreatment and saccharification processes, the development and validation of a rapid, simple, and effective analytical methodology for the simultaneous determination of 5-HMF and furfural in lignocellulosic biomass that underwent acid saccharification using high-performance liquid chromatography (HPLC) with photodiode array (PDA) detection is presented. It is important to note that this method may be applicable to biomass that underwent pretreatment prior to enzymatic saccharification.

## 2 Results and Discussion

### 2.1 Development and Validation of the Method

#### 2.1.1 Selectivity

Under the optimized chromatographic conditions, the analytes were effectively separated from other matrix components within a total run time of 12 min in isocratic elution. Notably, the presence of the analytes in the matrix was confirmed with good resolution and without the need for additional sample preparation, highlighting the practicality and efficiency of the proposed method (Figure 2). The number of theoretical plates calculated for the analyte peaks (Table 1) in the solvent and in the matrix did not vary significantly when compared to each other. Therefore, it can be stated that there was no significant interference from the matrix in the chromatographic separation of the target compounds. Furthermore, although the peaks presented an asymmetry factor > 1.0, all were significantly low and considered within the ideal range for quantitative chromatographic analysis (<2.0).

**Figure 2:**
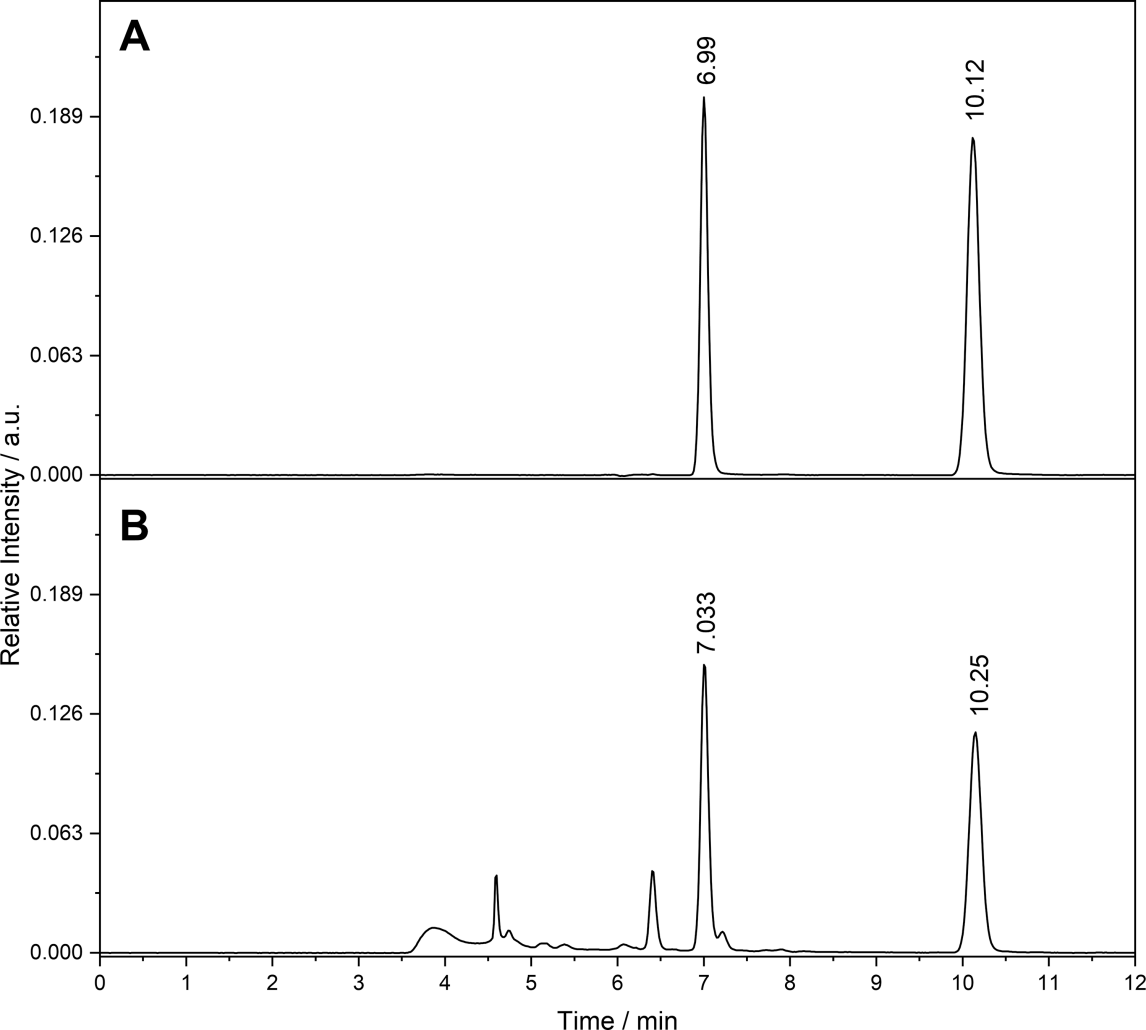
Chromatograms at 277 nm of (A) standards and (B) pure matrix.

**Table 1:** Chromatographic Parameters Calculated for the Standard Analytes and the Matrix

Analyte	λ_max_ (nm)	*t*_r_ (min)	*N* standard	*N* matrix	*A*_sym_ standard	*A*_sym_ matrix
5-HMF	285.0	7.029	28154.2	28029.0	1.214	1.386
furfural	277.2	10.22	22773.8	22782.3	1.141	1.040

In Figure 3, the UV-PDA spectra of the pure analytes and in the matrix are shown. It can be observed that the spectra in the matrix are identical in peak position and shape (except for intensity), indicating effective separation and reaffirming the absence of interferents at these retention times. Furthermore, the stability of the peak at other wavelengths also suggests this (Appendix 1). The maximum absorption λ slightly varies for each analyte, with 5-HMF at 285 nm and furfural at 277 nm. In both cases, these bands illustrate characteristic π → π* transitions of carboxylic groups. These compounds also exhibit a secondary, lower-intensity band around 230 nm, attributed to *n* → π* transitions, in the carboxylic group.^17^

**Figure 3:**
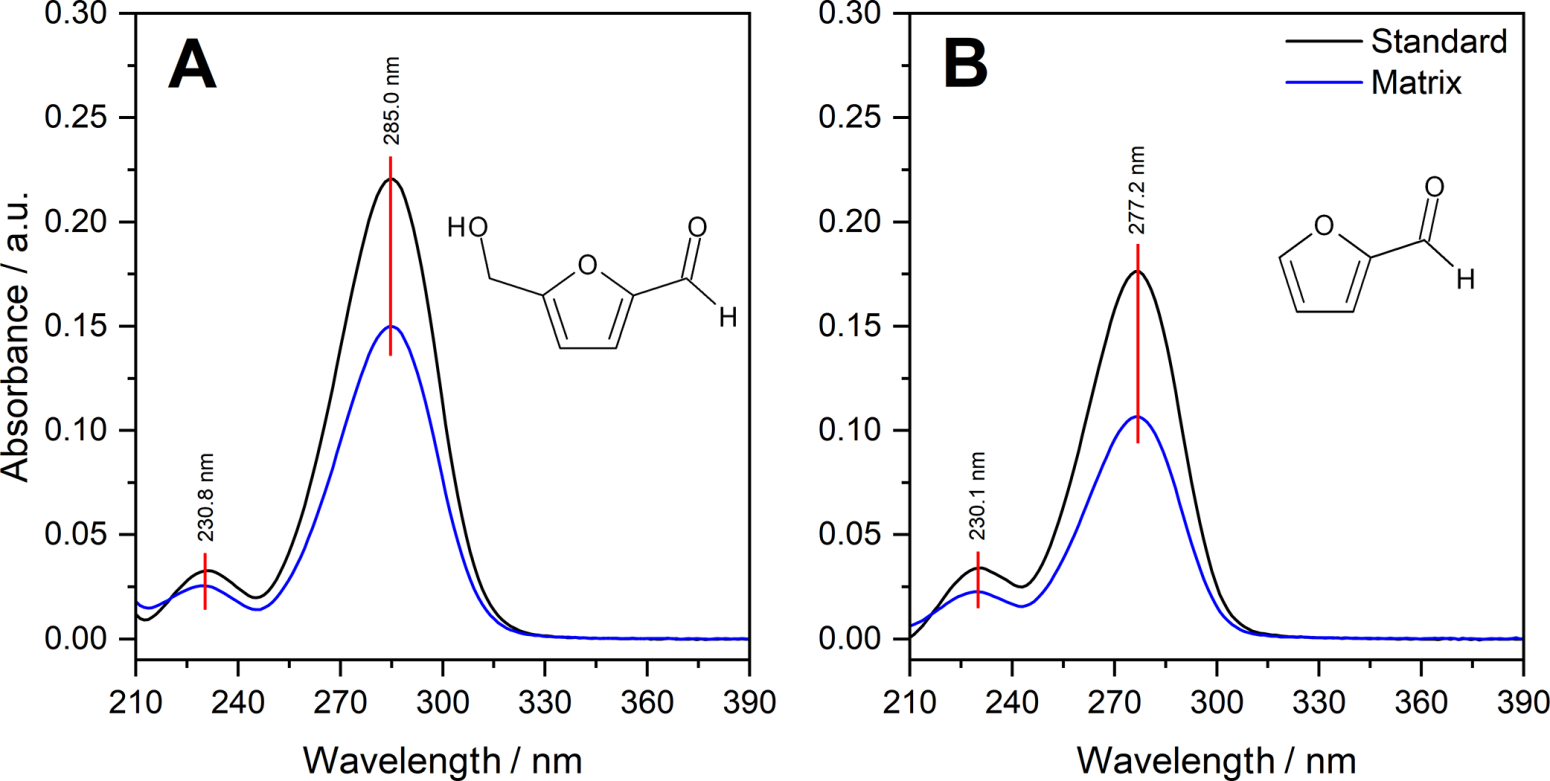
Ultraviolet spectra of the standard analytes and in the matrix (A) 5-HMF (B) furfural.

#### 2.1.2 Linearity

The detection range for the analytes was 50.0–0.1 μg·mL^–1^ for 5-HMF and 25.0–0.1 μg·mL^–1^ for furfural, values at which the concentration of the analytes as a function of integral peak area remained linear and covered the concentration in the samples studied. The chromatograms of the analytical curves are shown in Figure 4A,4B, and their analytical curves are shown in Figure 5A,B.

**Figure 4:**
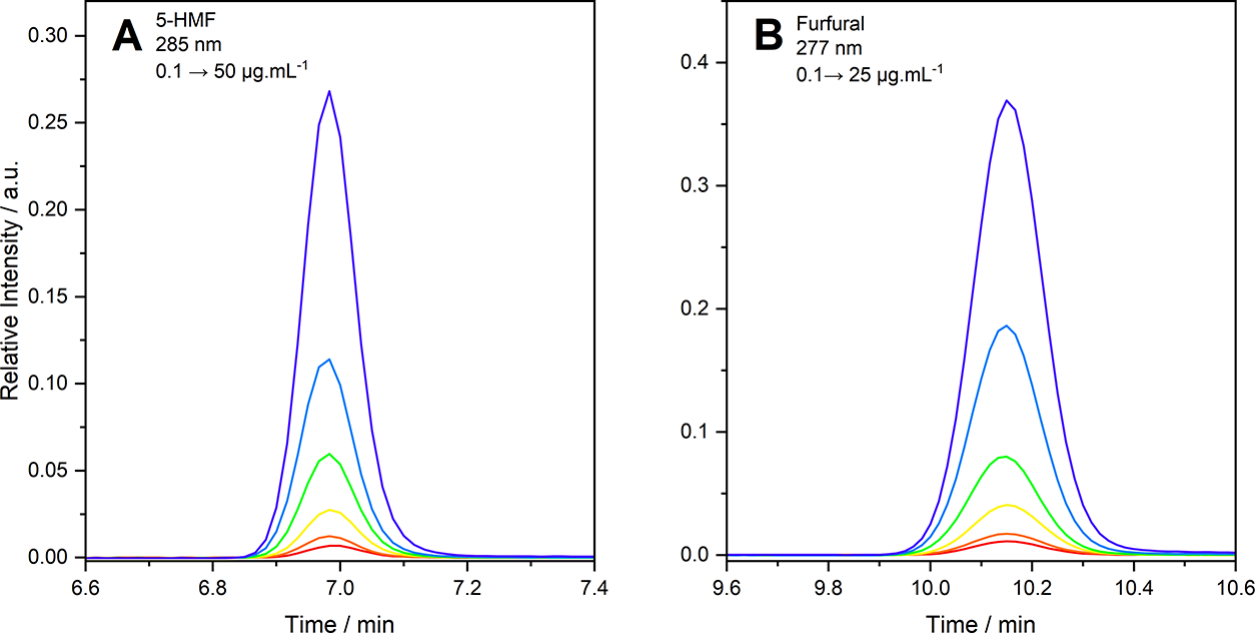
Chromatographic peaks of the analytical curves in (A) 5-HMF (λ = 285 nm) and (B) furfural (λ = 277 nm).

**Figure 5:**
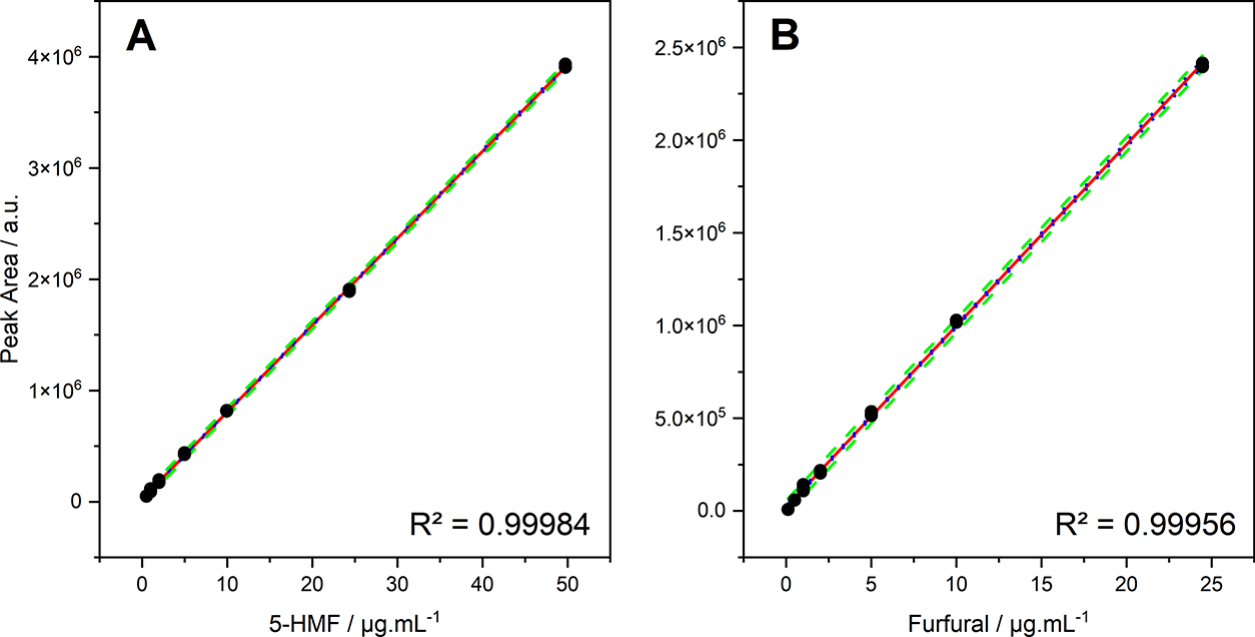
Analytical curve of the standards of (A) 5-HMF (285 nm) and (B) furfural (277 nm).

The linearity of the method was evaluated based on the linear regression models obtained from the analytical calibration curves, as presented in Table 2. The adjusted *R* ^2^ values, greater than 0.999 for both analytes, indicate an excellent fit of the model, explaining more than 99% of the variability of the experimental data. Furthermore, the statistical significance of the linear regression models was confirmed by ANOVA (Table 3), and the regressions showed p-values lower than 0.05, demonstrating that the variation in the response variables is indeed explained by the variation in analyte concentration and not due to random error. To reinforce the validity of the regression models, the normality of the standardized residuals was assessed using the Shapiro–Wilk test. The residuals were normally distributed for both 5-HMF (*W* = 0.965; *p* > 0.05) and furfural (*W* = 0.954; *p* > 0.05), indicating that the assumptions of the regression analysis were satisfied. These results confirm that the proposed method demonstrates excellent linearity, essential for ensuring the reliability of quantification across the evaluated concentration ranges.

**Table 2:** Regression Coefficients and Statistics^a^

Data	Intercept	SE	Slope	SE	*R* ^2^
5-HMF	2.715 E^4^	4.685 E^3^	7.805 E^4^	2.194 E^3^	0.99984
Furfural	1.992 E^4^	4.703 E^3^	9.794 E^4^	4.610 E^2^	0.99956

a SE, standard error.

**Table 3:** Analysis of Variance of the Regression Models (α = 0.05)^a^

Model	Variation	DF	SS	MS	*t*-value	*p*-value
5-HMF	Regression	1	3.604 E^13^	3.604 E^13^	1.266 E^5^	0
	Error	19	5.409 E^9^	2.847 E^8^		
	Total	20	3.604 E^13^			
Furfural	Regression	1	1.334 E^13^	1.334 E^13^	45126.961	0
	Error	19	5.618 E^9^	2.957 E^8^		
	Total	20	1.335 E^13^			

a DF, degrees of freedom; SS, sum of squares; and MS, mean squares.

#### 2.1.3 Limits of Detection and Quantification

The values of LOD and LOQ were calculated from the regression data. Both the LOD and LOQ values were low and within the expected range for the samples. For 5-HMF, de LOD = 0.1981 and LOQ = 0.6002 μg·mL^–1^ (ppm), and for furfural, the LOD = 0.1585 and LOQ = 0.4802 μg·mL^–1^. These results demonstrate that the method is sensitive enough to detect and quantify low concentrations of both 5-HMF and furfural in biomass samples. For 5-HMF, the method reaches concentrations well below the level reported to inhibit 32% of S. cerevisiae activity of CO_2_ conversion (4.0 mg·mL^–1^), as described by Taherzadeh et al.,^8^ and also below the regulatory limits established for food-derived samples such as honey (40–80 mg.kg^–1^ or ppm).^18^

When compared to recent methodologies in the literature, as shown in Table 4, the proposed method stands out in key analytical parameters. It achieves one of the lowest LOD and LOQ values for both 5-HMF and furfural reported recently, surpassing even methods that rely on internal standards.^18^ Unlike approaches that require labor-intensive and costly pretreatment steps, such as SPE^19^ or QuEChERS,^20^ this method allows for direct analysis of the sample, drastically reducing preparation time, cost, and potential analyte loss. Furthermore, its short total run time (12 min) is notably faster than those reported by Li et al.^16^ (28 min) and Alper^19^ (30 min), offering time efficiency for both research and quality control settings, making it more suitable for routine analytical workflows. The wide linear range and excellent precision strengthen its applicability for both low- and high-concentration scenarios in biomass hydrolysates.

**Table 4:** Comparison of Analytical Parameters for the Determination of 5-HMF and Furfural in Biomass and Its Derivatives Using Different HPLC-Based Methods Reported in the Recent Literature^a^

Reference	Method	Elution	Run time (min)	Analyte	Linear range (μg·mL^–1^)	LOD (μg·mL^–1^)	LOQ (μg·mL^–1^)
Li et al. 2017 ^16^	HPLC-UV	isocratic MeOH:H_2_O (20:80)	∼28	5-HMF	10–500	2.00	7.00
				furfural	10–500	3.00	9.00
Godoy et al. 2022 ^18^	HPLC-DAD, FDCA as internal standard	isocratic trisodium citrate buffer (pH 2.5)	15	5-HMF	3.78–26.48	0.300	4.45
				furfural	0.048–2.88	0.082	1.12
Alper, 2025 ^19^	SPE-HPLC-UV	MeOH:H_2_O (18:72)	30	5-HMF	0.25–500	19.8	60.1
				furfural	0.25–500	16.6	50.4
Dos Santos et al. 2025 ^20^	QuEChERS-HPLC-UV	isocratic MeCN:H_2_O (0.1% TFA) (25:75)	∼15	5-HMF	1.25–12.5	0.4001.30	1.35^b^ 3.93
				furfural	1.25–12.5	0.3500.340^b^	1.07^b^ 1.02^c^
This work	HPLC-PDA	isocratic MeCN:H_2_O (1% HCO_2_H) (40:60)	12	5-HMF	0.1–50.0	0.198^c^	0.600
				furfural	0.1–25.0	0.158	0.480

a Values converted from mmol·L^–1^ to μg·mL^–1^.

b Semisolid matrix from brewery spent grain’s hydrolysate.

c Liquid matrix from brewery spent grain’s hydrolysate; DAD, diode array detector, similar to PDA; FDCA, 2,5-furandicarboxylic acid; SPE, solid phase extraction pretreatment method; QuEChERS, quick, easy, cheap, effective, robust, and safe pretreatment method; and TFA, trifluoroacetic acid.

Together, these features make the method not only analytically robust and sensitive but also highly practical and scalable for routine analysis and industrial applications. It provides a strategic advantage for biofuel research and production by offering a simple, fast, and cost-effective solution for monitoring key degradation products, thus contributing directly to the advancement of sustainable biomass processing technologies.

#### 2.1.4 Accuracy and Precision

The accuracy of the method was assessed through recovery studies using the standard addition method, given the inability to obtain an analyte-free matrix. As shown in Table 5, recoveries ranged from 100.7 to 104.9% for 5-HMF and from 97.4 to 100.4% for furfural, all values within the acceptable criteria (80–110%) established by validation guidelines.^21^

**Table 5:** Recovery Percentages and RSD for Repeatability and Intermediate Precision

Analyte	Fortified sample (μg·mL^–1^)	Recovery (%)	Repeatability (RSD %)	Intermediary precision (RSD %)
5-HMF	2.460	100.7 ± 0.5828	1.255	1.143
	19.70	104.9 ± 1.578	3.125	2.650
	39.40	104.1 ± 7.385	2.813	1.970
Furfural	0.201	97.54 ± 1.424	1.388	2.050
	10.05	100.4 ± 0.7063	2.341	1.883
	20.10	97.71 ± 5.134	11.89	3.409

Precision was evaluated by repeatability (intraday) and intermediate precision (interday), also presented in Table 5. In all cases, RSD values were ≤15%, confirming that the method is precise, accurate, and reproducible. These results demonstrate the method’s reliability across different conditions and concentration levels. Additionally, compared to other methods reported in the literature,^16,18,20^ the developed method exhibits similar or superior performance in terms of recovery and precision, highlighting its versatility and robustness for monitoring 5-HMF and furfural in complex lignocellulosic matrices.

#### 2.1.5 Robustness

To evaluate the robustness of the method, small changes were made to the optimized chromatographic parameters to assess whether the method’s response under varying analytical conditions is reproducible. The results are summarized in Table 6. The method proved robust against minor changes in the mobile phase composition, as no statistically significant differences (*p* > 0.05) were observed in the mean results for either analyte. However, a significant difference (*p* < 0.05) was observed for 5-HMF under slight variations in flow rate, suggesting that this analyte’s quantification may be sensitive to flow changes. It is important to note that this does not necessarily imply a large practical difference but rather that the observed difference is unlikely to be random. For furfural, no statistical difference was observed (*p* > 0.05).

**Table 6:** Evaluation of the Method’s Robustness

Method	Level	5-HMF (μg·mL^–1^)	*t*-value	*p*-value	furfural (μg·mL^–1^)	*t*-value	*p*-value
Proposed		23.70 ± 0.2165			1.094 ± 0.2528		
Flow change	1.9	24.93 ± 0.0147	–10.40	0.009	0.9593 ± 0.0142	0.977	0.432
	2.1	22.78 ± 0.0340	7.057	0.019	0.8603 ± 0.0202	1.485	0.273
MeCN % change	38	23.84 ± 0.0433	–0.895	0.465	0.9052 ± 0.0253	1.261	0.334
	42	23.72 ± 0.2817	–0.138	0.902	0.8918 ± 0.0180	1.489	0.275

Overall, the method’s robustness ensures that it can be reliably applied in real-world conditions, even with slight operational adjustments, making it a practical tool for routine monitoring in biomass conversion processes. This robustness also enhances its potential for use in large-scale applications, where minor variations in the process parameters are common.

### 2.2 Real Sample Analysis

The methodology developed and validated was applied to 43 samples of H. brasiliensis hydrolyzed lignocellulosic biomass obtained from different acidic hydrolysis processes. According to Figure 6, the quantification results showed that the levels of 5-HMF in the samples ranged from 241.36 to 31.036 μg·mL^–1^, while furfural ranged from 0.177 (<LOQ, >LOD) to 22.315 μg·mL^–1^. These variations reflect not only the specific characteristics of the biomass but also the influence of the hydrolysis process used.

**Figure 6:**
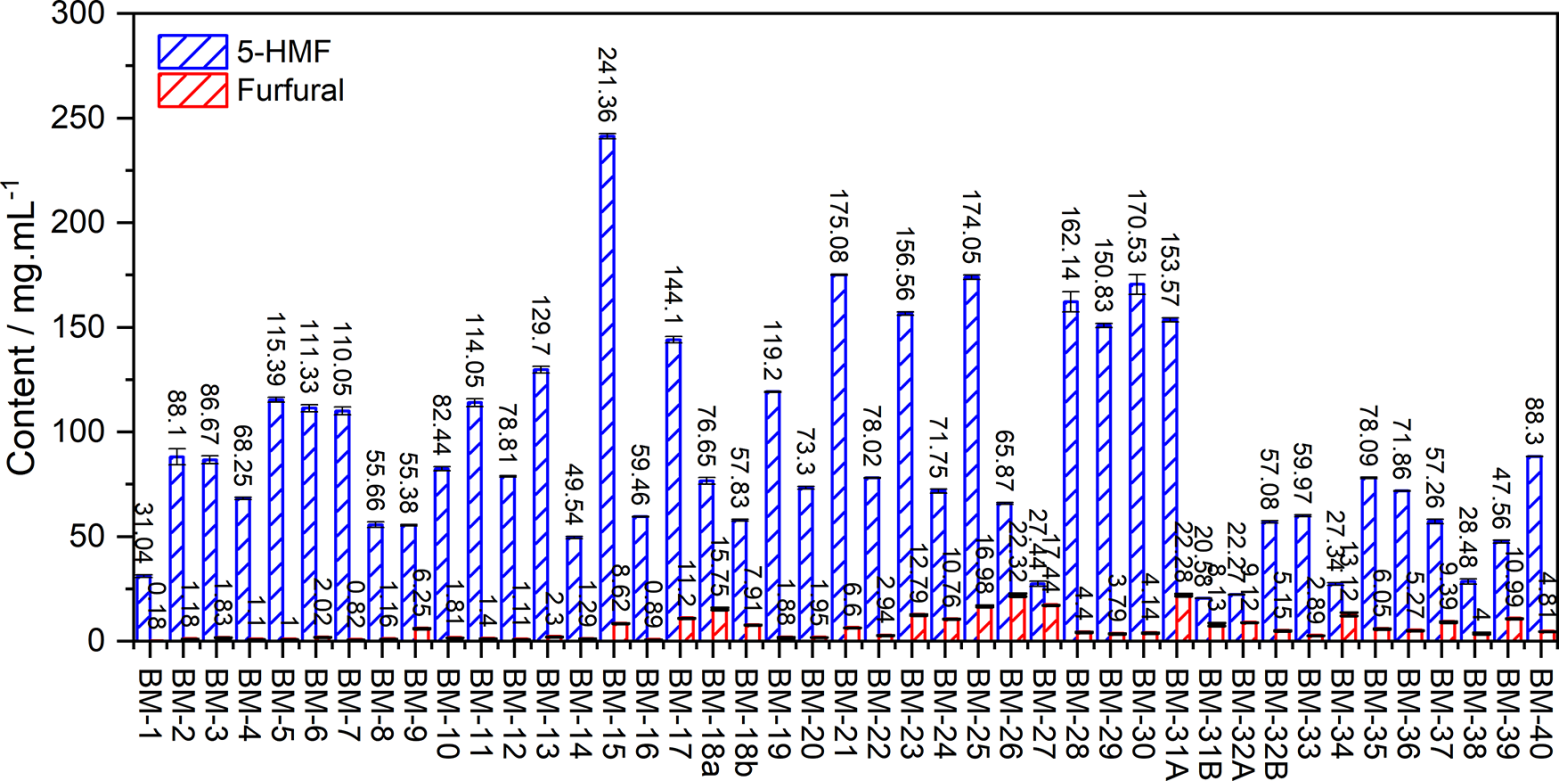
Content of 5-HMF and furfural in the hydrolyzed biomass samples.

In general, the samples exhibited significantly lower concentrations of furfural, suggesting a relatively low hemicellulose content in H. brasiliensis biomass, contrasted by higher 5-HMF levels, which are indicative of greater cellulose and starch content. According to the study by Riyaphan et al.,^22^ H. brasiliensis wood, as a comparison, presents a holocellulose content ranging from 68.0 to 73.0%, composed of approximately 38.3–42.0% α-cellulose and 29.7–32.8% hemicellulose, along with a relatively low lignin content (18.1–21.3%). These compositional characteristics are consistent with the analyte profile observed in this study. Furthermore, variations in the hydrolysis methods, such as acid concentration, temperature, and reaction time, directly influence the formation of these dehydration products.

The application of the developed method not only demonstrated its robustness and applicability but also highlighted its potential as a tool for optimizing hydrolysis conditions, aiming to minimize the formation of undesirable byproducts and, consequently, improve biomass conversion efficiency and bioethanol yields.

## 3 Conclusions

This study presents a fast, sensitive, and validated method for the simultaneous quantification of 5-HMF and furfural in hydrolyzed lignocellulosic biomass. It achieved low LODs and LOQs (0.1981/0.6002 μg·mL^–1^ for 5-HMF; 0.1585/0.4802 μg·mL^–1^ for furfural), with excellent precision and accuracy (RSD < 15%). Applied to 43 real samples from hydrolyzed H. brasiliensis lignocellulosic biomass, the method enables direct analysis without complex pretreatment steps, significantly reducing operational time and costs. Its simplicity, robustness, and reliability make it highly suitable for routine monitoring and process optimization, offering valuable support for improving biomass conversion efficiency and advancing bioethanol production initiatives.

## 4 Methods

All solvents and standards used are HPLC purity grade (Table 7). All solutions were prepared using ultrapure water obtained from a Milli-Q filtration system from Merck Millipore. All glassware used in this study was subjected to constant washing and cleaning using ultrapure water and methanol in an ultrasonic agitation system for 15 min.

**Table 7:** Solvents and Standards Used and Their Respective Sources and Purities

Substance	Chemical formula	Source	Purity (%)
Acetonitrile	CH_3_CN	Tedia	≥99.9
Formic acid	H_2_COOH	Sigma-Aldrich	98–99
5-(hydroxymethyl)-2-furfuraldehyde	C_6_H_6_O_3_	Sigma-Aldrich	99
2-furfuraldehyde	C_5_H_4_O_2_	Vetec	≥98
Methanol	H_3_COH	Tedia	>99

### 4.1 Biomass Processing

The biomass was obtained from H. brasiliensis Müll.Arg. (Euphorbiaceae), colloquially known as “seringueira” or “rubber tree”, provided by Kaiser Agro Florestal LTDA. The biomass underwent an oil extraction process via mechanical pressing. The resulting defatted solid residue was selected as the raw material for subsequent acid hydrolysis. The hydrolysis was carried out in an autoclave to ensure precise temperature and pressure control. Different conditions were tested for each sample by varying the concentration of the acid mixture (H_2_SO_4_, H_3_PO_4_, and HCl), the reaction temperature, hydrolysis exposure time, and biomass concentration. After hydrolysis, residual acids were neutralized with sodium hydroxide (NaOH). The resulting mixture was then brought to a predefined volume by using a volumetric flask for standardization and filtered through filter paper.

As a key differential of this study, the H. brasiliensis hydrolyzed lignocellulosic biomass samples underwent no complex or costly pretreatment methods for HPLC-DAD analysis. Instead, the samples were simply diluted and filtered using a 0.22 μm PTFE membrane filter to remove the residual solid particles. The treated samples were then transferred to vials, ready for direct instrumental analysis, or could be stored under controlled conditions for further use.

### 4.2 High-Performance Liquid Chromatography (HPLC-PDA)

All data were acquired using the Waters 600 high-performance liquid chromatography (HPLC) system with a quaternary solvent pumping system coupled to a photodiode array (PDA) detector. The column used was the Phenomenex Luna C18 (250 × 10 mm) 5 μm, 100Å. The mobile phase used for isocratic elution was ultrapure water + 1% formic acid/acetonitrile (60:40), with a flow rate of 2 mL·min^–1^ and a column temperature of 35 °C. The injection volume loop was 20 μL.

The analytes were monitored at 285 nm for 5-HMF and 277 nm for furfural.^16,23^ The concentration range studied, where the area under the chromatographic peak was linearly proportional to the analyte concentration, was 0.1–50.0 μg·mL^–1^ for 5-HMF and 0.1–25.0 μg·mL^–1^ for furfural.

### 4.3 Analytical Validation

The entire statistical validation of the proposed analytical methods was evaluated using the parameters recommended by INMETRO (Instituto Nacional de Metrologia, Qualidade e Tecnologia—National Institute of Metrology, Quality and Technology),^21^ which are Selectivity, Linearity, Limit of Detection and Quantification, Repeatability, Intermediate Precision, and Accuracy. Robustness, an optional criterion, was also assessed.

To evaluate the specificity and selectivity of the method, chromatograms of the analytes in standard solution and the matrix, already rich in analytes, were compared. The asymmetry factors of the peaks (*A*_sym_) were calculated by using eq 1 and compared. Here, *A*_10%_ is the peak width on the left side at 10% height, and *B*_10%_ is the peak width on the right side at 10% height. Similarly, the number of theoretical plates (*N*) was determined and compared to check whether the matrix interferes with the separation of the analytes, according to eq 2, where *t*_r_ is the analyte retention time and *W*_1/2_ is the peak’s half-width.

1 $$A_{\mathrm{sym}}=\frac{B_{10\%}}{A_{10\%}}$$

2 $$N=5.54{\left(\frac{t_{r}}{W_{1/2}}\right)}^{2}$$

The linearity was assessed by fitting the data to the linear model and analyzing its residuals through the analysis of variance (ANOVA) (α = 0.05). The Limit of detection (LOD) and quantification (LOQ) were calculated according to eqs 3 and 4, respectively. Where SD_*a*_ corresponds to the standard deviation of the linear coefficient and *b* is the angular coefficient, both from the regression line. To validate the parametricity of the data, the normality of the residuals was evaluated using the Shapiro–Wilk test.

3 $$\mathrm{LOD}=\frac{3.3{\mathrm{SD}}_{a}}{b}$$

4 $$\mathrm{LOQ}=\frac{10SD_{a}}{b}$$

The accuracy of the method was determined by the recovery percentage (*R*_%_) of the analyte in a fortified sample with a known concentration, according to eq 5, where *c*_1_ is the concentration obtained from the fortified sample, *c*_2_ is the concentration of the nonfortified sample, and *c*_real_ is the real added concentration. Recovery was evaluated at three different concentrations: low (0.5 and 0.1 μg·mL^–1^ for 5-HMF and Furfural, respectively), intermediate (20 and 10 μg·mL^–1^), and high (40 and 20 μg·mL^–1^), within the working range. According to INMETRO^21^ criteria, within the studied range (1 ppm–100 ppb), the average recoveries should be between 80 and 110% to be classified as acceptable.

5 $$R_{\%}=\frac{c_{1}-c_{2}}{c_{\mathrm{real}}}\times 100$$

6 $$\mathrm{RSD}=\frac{\mathrm{SD}}{\mathrm{x̅}}\times 100$$

The precision of the method was evaluated through intermediate precision and repeatability. Repeatability involves recovery tests performed at different times on the same day (intraday), while intermediate precision is assessed over three different days (Interday). Precision was expressed in terms of the relative standard deviation (RSD), according to eq 6, where SD is the standard deviation of the measurements and *x̅* is the average determined concentration. According to INMETRO^21^ criteria, based on the studied concentration range, RSD values should be ≤15% to be considered acceptable.

To assess robustness, the method’s resistance to small variations in operational parameters, and to indicate the stability and reliability of the method, small variations were Applied, univariately, in the mobile phase composition (+2 and −2% of MeCN) and flow rate (+0.1 and −0.1 mL·min^–1^). The method’s ability to determine the concentration in the sample was evaluated by comparing the *x̅* of the modified method with the *x̅* of the proposed standard method using a paired *t*-test.

## Supplementary Material



## Notes

Chromatograms of one sample matrix in different wavelengths (PDF)

## Abbreviations

HPLC: High recision Liquid Chromatography
PDA: Photodiode Array
5-HMF: 5-(hydroxymethyl)-2-furfuraldehyde
INMETRO: Institute of Metrology and Standardization
LOD: Limit of Detection
LOQ: Limit of Quantification
RSD: Relative Standard Deviation.
