# Supplementary material for: Simultaneous Determination of Yeast Inhibitors 5‑HMF and Furfural in Hydrolyzed Lignocellulosic Biomass using HPLC-PDA
Source: ACS Omega. 2025 Jun 2;10(22):23827–35. doi: 10.1021/acsomega.5c03283 (PMC12163807; doi:10.1021/acsomega.5c03283)
Supplement: Supplementary file 1 [file ao5c03283_si_001.pdf]

SIMULTANEOUS DETERMINATION OF YEAST INHIBITORS 5-HMF AND FURFURAL IN HYDROLYZED LIGNOCELLULOSIC BIOMASS USING HPLC-PDA

JHONATAN M. P. ROCHA[*a]; GIOVANO TOCHETTO[b]; ANDRÉ L. GALLINA[b]; DAIANE F. FERREIRA[a].*

[a] Universidade Estadual do Centro-Oeste *Campus* CEDETEG, Chemistry Department, Elio Antônio Dalla Vecchia Avenue, 838, Vila Carli CEP 85040-167, Guarapuava, Paraná, Brazil.

[b] Universidade Estadual do Centro-Oeste *Campus* CEDETEG, Post-graduate Program in Bioenergy, *Campus* CEDETEG, Elio Antônio Dalla Vecchia Avenue, 838, Vila Carli CEP 85040-167, Guarapuava, Paraná, Brazil.

ASSOCIATED CONTENT

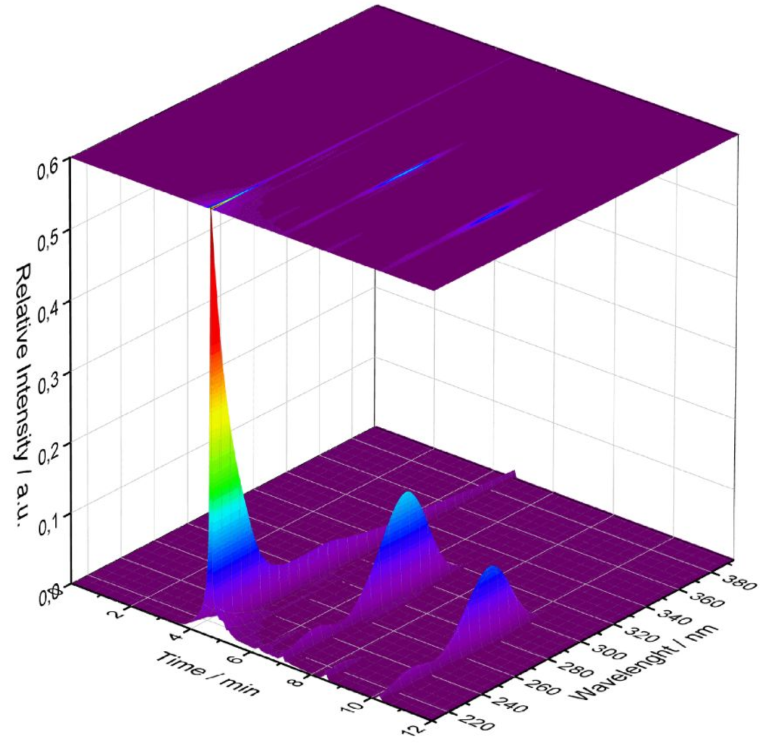

Appendix 1. Chromatograms of one sample matrix in different wavelengths
